# Supplementary material for: Toward a theory of evolution as multilevel learning
Source: Proc Natl Acad Sci U S A. 2022 Feb 4;119(6):e2120037119. doi: 10.1073/pnas.2120037119 (PMC8833143; doi:10.1073/pnas.2120037119)
Supplement: Supplementary File [file pnas.2120037119.sapp.pdf]

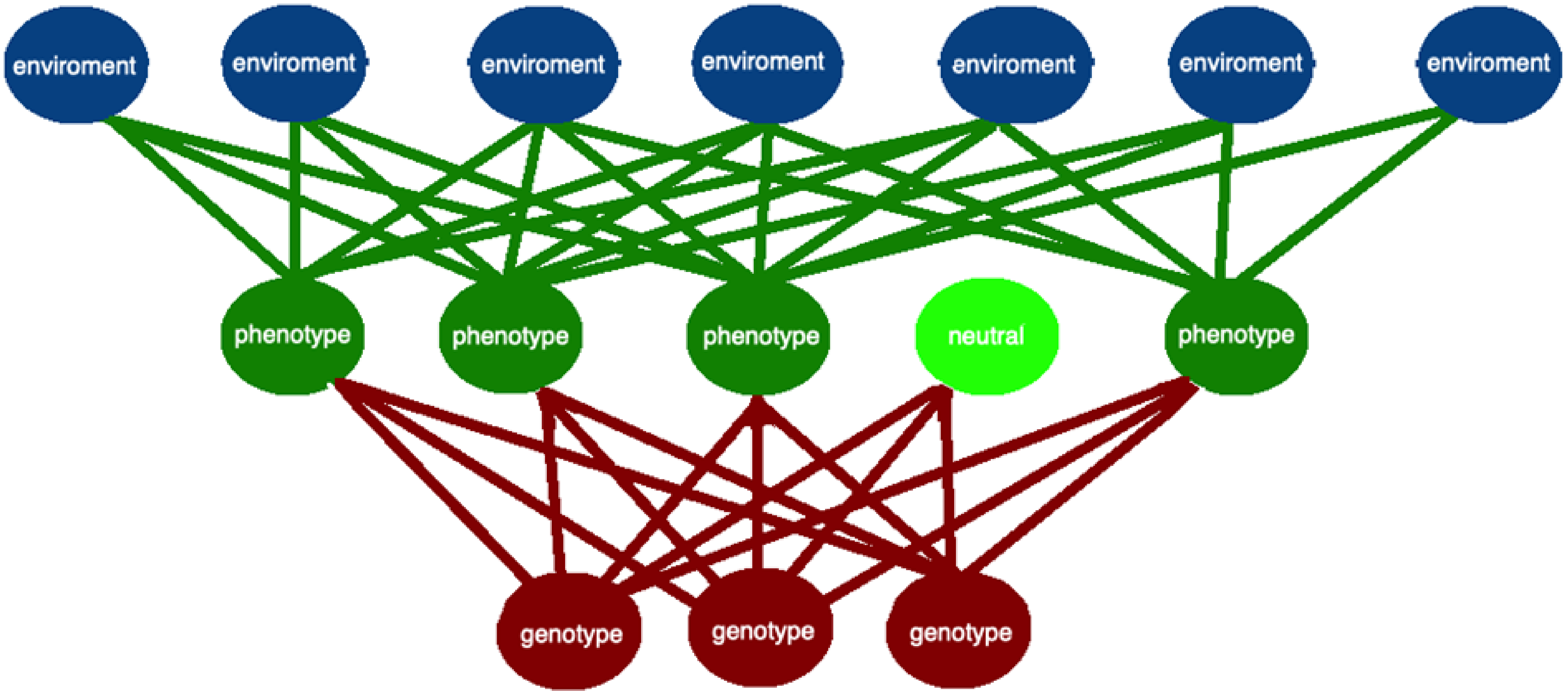

Figure S1. **Neural network with three layers**.
Non-trainable environmental variables (blue nodes), non-trainable organism variables (red and green nodes);
trainable, intermediate-changing, adaptive phenotypic variables (dark green nodes and links);
trainable, slow-changing (near constant), genotype variables (red nodes/links); and neutral variables (light green node).

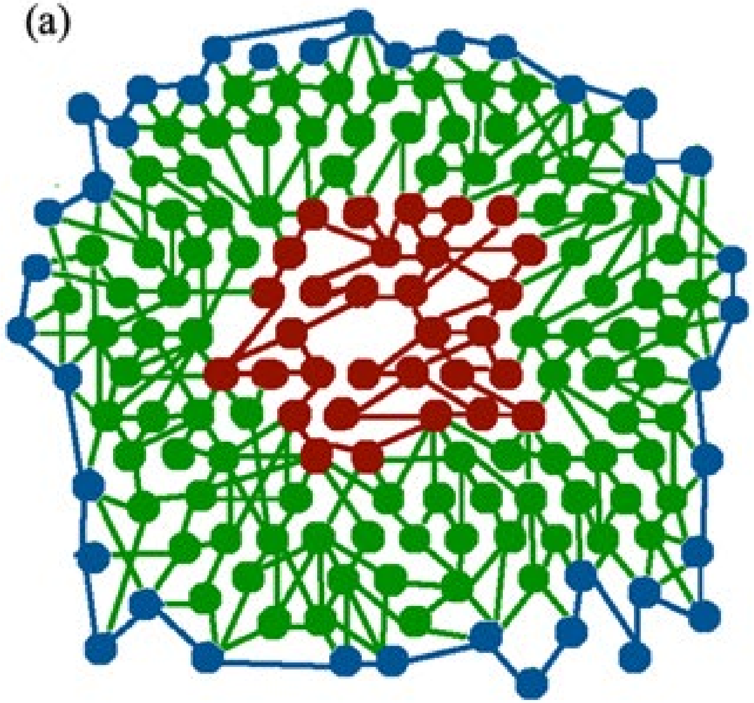

Figure S2. **Separation of variables in a learning system depending on the time scale.**
Three states of a learning system are shown, with fast-changing environmental variables (blue nodes and links), intermediate-changing trainable variables (green nodes and links), and slow-changing trainable variables (red nodes and links) observed on three different time-scales, from the shortest to the longest: (a) (b) (c).

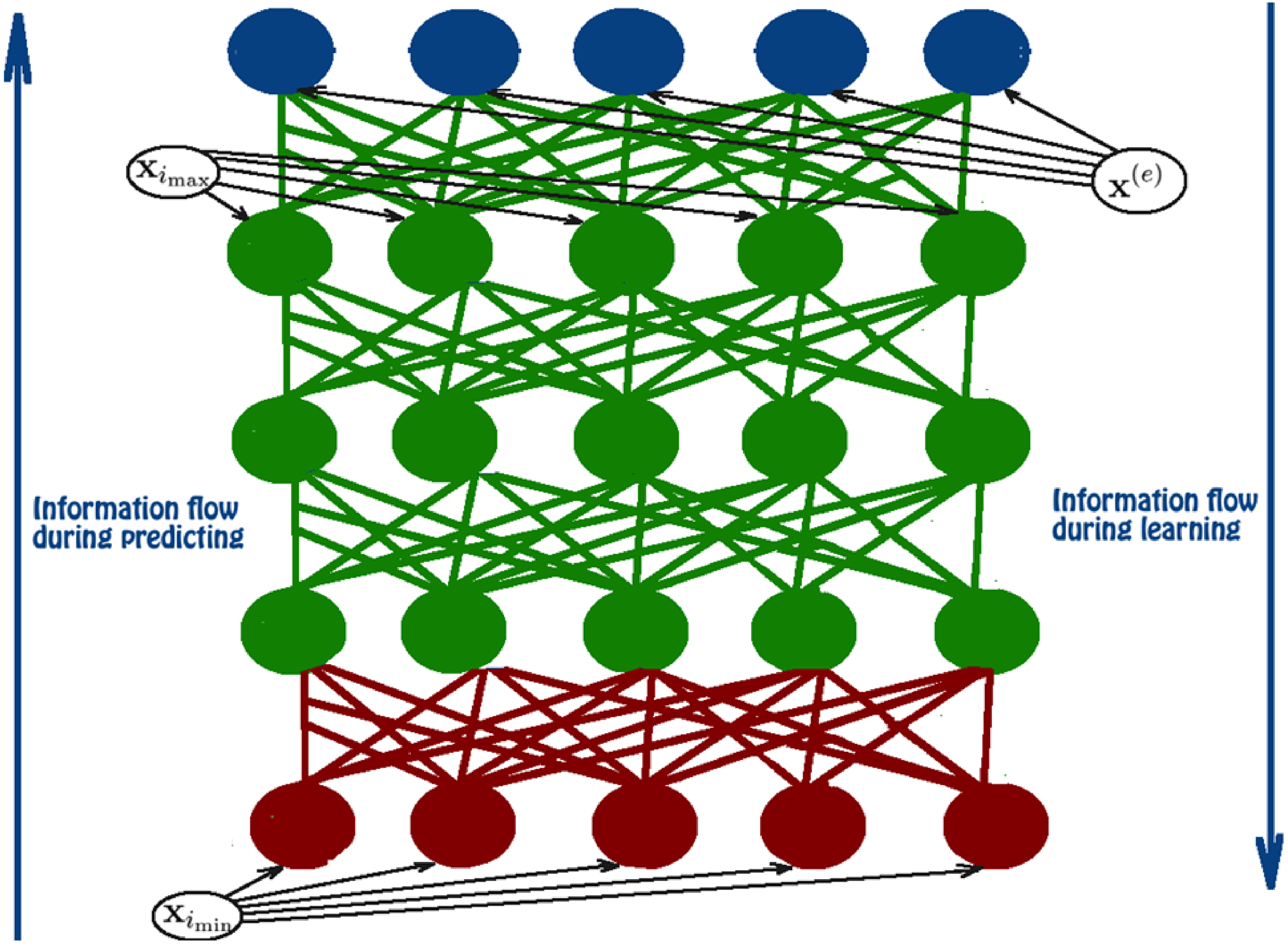

Figure S3. **Asymmetrical information flow involved in learning and predicting the environment by evolving systems: generalized Central Dogma of molecular biology**.
